# Supplementary material for: Moesziomyces antarcticus MMF1 Has a Role in the Secretion of Mannosylerythritol Lipids
Source: Microorganisms. 2025 Jun 24;13(7):1463. doi: 10.3390/microorganisms13071463 (PMC12299085; doi:10.3390/microorganisms13071463)
Supplement: Supplementary file 1 [file microorganisms-13-01463-s001.zip › microorganisms-3670129-supplementary.pdf]

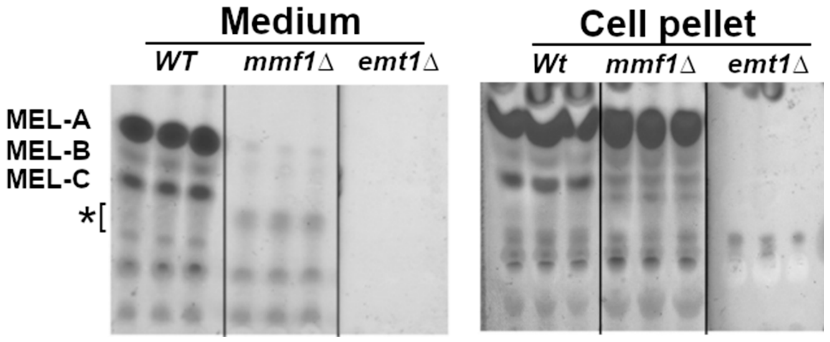

Figure S1. ***M. antarcticus*** **strains deficient in** ***MMF1*** **display a defect in MEL secretion**. Three biological replicates of strains *MMF1 (WT)*, *mmf1::HYGRO (mmf1Δ)* and *emt1::HYGRO (emt1Δ)* were cultured in MEL production medium supplemented with 4% glucose as a carbon source. Following three days of incubation, equivalent samples were collected from each culture and the cell pellets were separated from the culture medium prior to extraction of MEL. The MEL extracted from the culture medium (Medium) and cell pellets (Cell pellet) were separated by TLC detected with orcinol staining. The major MEL products MEL-A/B/C are indicated on the right. The position of an orcinol staining glycolipid species enriched in the *mmf1Δ* strains is indicated by the asterisk
